# The Community Structure of the European Network of Interlocking Directorates 2005–2010

**DOI:** 10.1371/journal.pone.0068581

**Published:** 2013-07-19

**Authors:** Eelke M. Heemskerk, Fabio Daolio, Marco Tomassini

**Affiliations:** 1 Department of Political Science, University of Amsterdam, Amsterdam, The Netherlands; 2 Department of Information Systems, University of Lausanne, Lausanne, Switzerland; 3 Department of Information Systems, University of Lausanne, Lausanne, Switzerland; Universidad Carlos III de Madrid, Spain

## Abstract

The boards of directors at large European companies overlap with each other to a sizable extent both within and across national borders. This could have important economic, political and management consequences. In this work we study in detail the topological structure of the networks that arise from this phenomenon. Using a comprehensive information database, we reconstruct the implicit networks of shared directorates among the top 300 European firms in 2005 and 2010, and suggest a number of novel ways to explore the trans-nationality of such business elite networks. Powerful community detection heuristics indicate that geography still plays an important role: there exist clear communities and they have a distinct national character. Nonetheless, from 2005 to 2010 we observe a densification of the boards interlocks network and a larger transnational orientation in its communities. Together with central actors and assortativity analyses, we provide statistical evidence that, at the level of corporate governance, Europe is getting closer.

## Introduction

Ever since the birth of the modern corporation, interfirm relations that tie firms together in networks of ownership and control have been in place. At the level of corporate governance, corporations are connected through shared board members (interlocking directorates), shared owners, and direct stock- holdings between firms. Although often depicted as atomic, individualistic disconnected market actors corporations are in fact deeply embedded in such networks. And in our days of ongoing financial, economic and cultural globalization it may come to no surprise that recent findings reveal that in the global network of corporate ownership a small group of corporations dominate. 40% of the control over economic value of Trans National Corporations in the world is held, through complicated ownership structures, by a group of 147 of these corporations [1]. What remains puzzling however is the enduring absence of a transnational network of corporate directors. Even in the closing decades of the 20th century international networks of interlocking directorates remained thin and best described as superstructures that rest on rather resilient national bases [2]. This observation leads to one of the main puzzles in corporate governance network research: to what extent is there a transnational network of interlocking directorates that extends beyond national bases.

Interlocking directorates, where two firms share at least one director, build a social network that mutually connects the top decision making bodies in our economies and its directors. A lively illustration of the interconnectedness of corporate boards in the USA reminds that a corporate governance reform (or a rumor) discussed at the a Chase Manhattan Banks board meeting in January could make its way via face-to-face contact to the boards of 97% of the largest USA corporations by May. If a virus would spread by handshake such a disease could infect almost the entire corporate in under half a year [3]. A naive but illustrative simulation of such a process has been provided in [4] for the case of Switzerland. A more refined model of the process of opinion formation and decision dynamics in the corporate board network of the US in 1999 has been presented in Battiston et al. [5]. Other investigations dealing with the Italian and American corporate board systems have been published in [6], [7]. The network of board interlocks is consequential because it serves as an opportunity structure for the spread of business practices [8], [9] reduces resource dependencies of firms [10], [11], and connects corporate control in an elite community of corporate directors [12], [13].

The emergence of transnational networks between corporate boards has long been anticipated as a sign of the globalization of capital [14]–[16]. However, it failed to emerge in practice. The 1970s did saw the creation of a minimal set of transnational interlocking directorates between business and finance spanning the Atlantic [14] but a follow up study showed that by the mid-1990s the international network has remained remarkably stable [2]. The absence has been attributed to cultural and institutional differences and to the enduring importance of national business communities for local elites [2], [17]. Also, it has been pointed out that in a social network such as that of corporate directors, geographical distance significantly increases the costs of maintaining ties that connect distant parts of the globe [18]. At the same time, corporate boards are nowadays increasingly nationally diverse [19], hinting at a shifting orientation of corporate directors from the national to the transnational. But does this already translate into transnational social networks as well? Empirical studies show that a small recent increase in transnational interlocking between 1996 and 2006 took place within Europe [20] and that a European corporate elite network seems to be emerging [21].

The aim of this paper is establish to which extent there is an (emerging) European network of interlocking corporate boards. In what follows we suggest a number of novel ways to explore the trans-nationality of the European business community, most notably through community detection algorithms and through introducing geographic assortivity. Our main research issue leads us to formulate four sets of sub questions that will guide the analysis in the following section. First of all we confirm previous studies that show that the network of interlocking directorates actually grows between 2005 and 2010 and that this is at least in part due to transnational interlocking. Second we consider the generative mechanisms that drive tie formation. On the one hand, we can expect to find traces of homophily, where well connected firms prefer to connect to other well connected firms. Homophily produces assortative mixing, where a contact between similar actors occurs at a higher rate than among dissimilar people [22]. (Note that this stands in contrast with preferential attachment, a common feature in complex networks but hardly applicable to social networks). Similar to this assortivity of degree, firms can also display homophily regarding the transnational network orientation of the boards. Thus, geographic assortivity reflects the process where transnationally connected firms prefer to connect to other well transnationally connected firm. The third sub question considers the central pillars in the network. Here we ask if the core of the network is confined to a set of firms within one country, or if it is of a more transnational composition. In addition the transnational character of the European network can be assessed by investigating how transnational the ego networks of the most central firms are. Fourth and finally, we suggest a novel way of using community detection algorithms to determine the transnational orientation of a given network of interlocking directorates. Locating communities within the network allows us to investigate if the European network is a superstructure upon national basis or (in part) a self-supporting elite social structure.

Together, these four sets of questions will allow us to determine the character of the emerging European network of interlocking directorates. Building on a recent study [17], [18], we compare the board network of the largest stock listed European firms (FTSE300) in 2005 and 2010 for their (community) structures.

## Methods

For the analysis, we have selected the largest European firms as listed in the Eurofirst top 300 as per market capitalization in the FTSE Developed Europe Index. This excludes firms from Eastern European countries, although several among them belong to the European Community. This should not be a problem as those countries have joined the Community recently. Some countries from the AELE such as Norway and Switzerland are also included. For each firm we recorded the country where its headquarters are located, the prevailing economic sector to which it belongs, as well as the composition of the board of directors [17]. The data have been checked for true duplicate names and for differently reported names (see also [17], [18]).

From the knowledge of which director sits in which board, one can build a bipartite network by assigning a node to each director and to each board. In the resulting graph 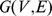 in which 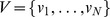 is the set of directors or companies, and 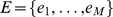 is the set of edges or links, the vertices can be partitioned into two disjoint sets 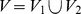, 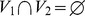, such that there are no edges 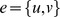 between vertices belonging to the same set: 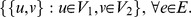 A link between a director and a board means that the director sits on that board. When two boards share the same director it is said that there is an *interlock*. *Multiple interlocks* are also possible, in which at least two directors of a board sit together on another board.

From the bipartite graph it is easy to obtain two derived graphs also called projections. One in which two directors are connected if they sit on the same board and another in which two boards are connected if they share a common director. In this work we focus on the boards weighted projection, where the weight 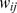 of a link 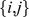 is the number of shared directors between firms 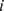 and 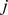. The boards graph has been produced for the years 2005 and 2010. In both cases the total graph is partitioned into a number of connected components, of which we have kept the giant ones for the analysis. In 2005 the giant component comprises 265 nodes out of 300, and in 2010 the number of nodes is 259.

On those graphs, we performed the following statistical analyses. In the first place, we measured the network density, which was defined as the ratio between the number of edges present in the networks and the number of edges of a complete graph with the same number of vertices, which can equally be expressed as 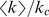 in terms of ratio between the average degree and the maximum possible degree. Then we have studied distance relationships in the networks as represented by the *Average Path Length* and *diameter*. The average path length was measured according to the formula 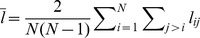 [23]. The diameter is the maximum among the shortest paths.

The *clustering coefficient* 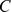, i.e.the propensity to form triangles that show *transitivity* in the network connectivity, is defined as the ratio of the number of closed paths of length two to the number of paths of length two [23]. Both the distance metrics as well as the clustering have been calculated for the unweighted graphs.

Furthermore, the degree distribution function 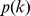 gives the probability that a randomly chosen vertex in the network has degree 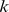. Here we use the complement 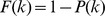 of the cumulative distribution function 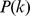, which is the integral (sum in our discrete case) of 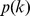 in a given 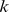 range. Since the board projection is weighted and each node 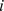 has a strength 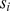 given by the sum of the weights of all links incident in 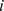, we also compute the cumulative strength distribution function 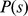.

As for actors' *centrality*, we have employed two measures: Bonacich's eigenvector centrality [24] and vertex betweenness centrality [25], both taking into account edge weights. In particular, for the latter, the length of an edge was assumed to be 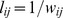. We discuss the substantive differences of these two measures in the empirical section.

We have studied *mixing patterns*, i.e. assortative or disassortative relationships for categorical data with Newman's assortativity coefficient 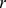 [26]. It measures the fraction of edges that run between vertices of the same type minus the expected fraction of such edges in a uniformly random graph with the same degree sequence.

Communities are, loosely speaking, groups of nodes in a network that are more strongly connected among themselves than with the remaining nodes (for an excellent review of this technically difficult topic see [27]). We have performed community detection on our graphs with the heuristic approaches of [28] and [29], taking the weights of links into account. The *modularity* metric [30] was then used as a merit function to assess the network partitioning suggested by the community finding algorithm. Modularity was also used to rank cluster formation based on criteria other than purely topological criteria such as economic sectors and geographical location. The modularity 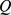 is defined as 
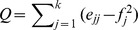
 where 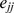 is the fraction of edges within community 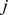, 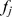 is the fraction of edges with one or both ends in community 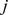 in a uniformly random network with the same degree sequence as the original one, and the summation runs over all 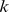 communities. However, modularity in itself is not always a witness of any meaningful community structure, as pointed out in [31]. To compare with a suitable null model, we have performed a Monte Carlo generation of 1000 instances of a randomized version of our data consisting in building graphs with the same degree sequence but with rewired edges [32], and shuffled edge weights. On these data we have computed the significance of observed modularity 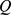 of the real networks.

All the data treatment and analysis has been carried out in R [33], for the most part with the “igraph” package [34].

## Results

In this section we present the results of the analysis. It first discusses the basic statistics of the network in the years 2005 and 2010, in order to confirm the general thrust of its evolution. Next the properties of assortativity and centrality are analyzed, after which we turn to an investigation of the community patterns.

### Basic Statistics

The largest corporations in Europe increase their network density through interlocking directorates from 2005 to 2010; the total number of edges increases by 13.7 percent (Table 1). Indeed average degree and network density both increase over time as well. Also, the European network of interlocking directorates is not scattered over disconnected subsets but organized in one large dominant component. In this the European network is similar to national networks of interlocking directorates [35]. The average distance in the network decreased from almost four to 3.44 and the diameter decreased from ten to nine. The network has indeed strengthened over time. Notably the clustering coefficient decreased somewhat. Although the drop is small, it does suggest that firms are increasingly interested to forge ties through shared directors with other firms that are not already in their direct neighborhood.

**Table 1:** Basic statistics of the largest connected components of boards projections.

Year|	*N*	*E*	*N/N*_k>0_	〈*k*〉	〈*k*〉/*k_c_*	*r*	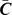	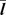	*d*
2005 | 2010 | Δ%|	265	850	0.97	6.42	0.024	0.34	0.29	3.92	10
	259	966	0.99	7.46	0.029	0.21	0.23	3.44	9
	−2.3	+13.7	+2.2	+16.3	+19.0	−38.3	−18.8	−12.2	−10.0

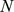
  = number of firms, 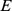  = number of edges, 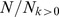 relative size of the largest connected component in the subset of firms having at least one connection, 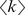  = average degree, 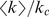  = graph density, 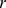  = degree assortativity, 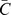  = average clustering coefficient, 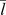  = average path length, 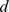  = diameter. All this measures apply to the unweighted graphs.

The degree distribution has an almost exponential decay which is slightly faster in 2010 than in 2005 (see the left-hand side of Figure S1 in File S1 for the details). This suggests that the network has grown essentially by random attachments of nodes rather than some sort of preferential attachment, and the distribution hasn't changed significantly between 2005 and 2010. However, looking also at the evolution of the mean degree (Table 1), we see that whereas boards are more interconnected on average in 2010, the maximum observed number of interlocks per board was higher in 2005 as the tail of the distribution is longer. The strength distribution has a behavior similar to the degree distribution except that the strength distribution has a slightly longer tail in 2010 (see the right-hand side of Figure S1 in File S1).

**Figure 1:**
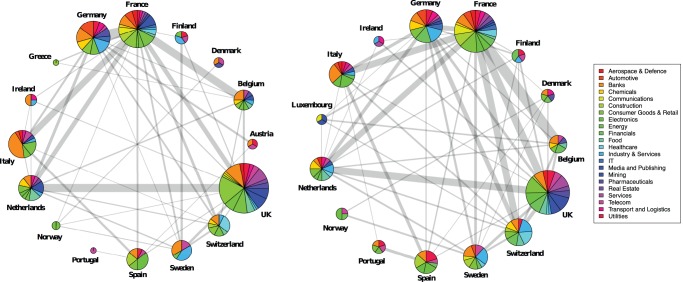
Board interlocks among EU countries in 2005 (Left) and 2010 (Right). The size of a node is proportional to the square root of the number of companies from that country, the thickness of an edge is proportional to the number of interlocks between the boards of companies from the connecting countries. Each node reports in a pie-chart the distribution of companies among the sectors listed on the right.

**Figure 2:**
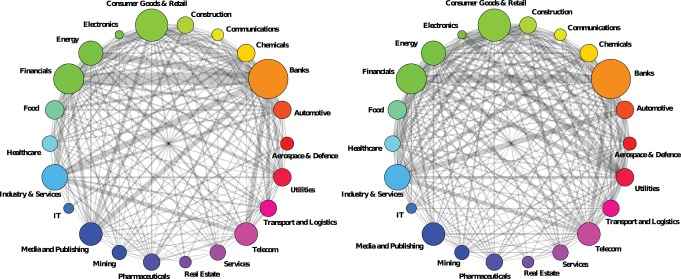
Board interlocks among sectors in 2005 (Left) and 2010 (Right). The size of a node is proportional to the square root of the number of companies in that sector, the thickness of an edge is proportional to the number of interlocks it represents.

Figures 1 and 2 represent the board interlocks across European countries and economic sectors, along with the distribution of sectors within countries. These graphs have been extracted from the board projection by grouping together firms belonging to the same country, and to the same economic sector respectively. Therefore, Fig. 1 displays transnational links only, whilst in Fig. 2 nodes represent firms from the same sector, no matter the country. We include sector in the analysis because we want to compare the geographic clustering of interlocking directorates with economic clustering within and between sectors. UK, France, and Germany are well represented in the sample, while small countries such as the Netherlands and Switzerland show relatively high levels of degree centrality: they are hubs in the network. The density of the country network increases from 0.3583 in 2005 to 0.4857 in 2010 implying an internationalization of the boards composition. When we consider the sectors, Banks, Consumer Goods & Retail, and Financials are the more frequent ones. The financial crisis reflects itself in the decreasing presence of banks over time. This can be qualitatively appreciated in Fig. 2 observing that the strength of several important links from the bank sector to other sectors has become weaker, as shown by the diminishing thickness of the respective edges. On the same note, we see that Utilities are on the increase. The very high density of the sector graph (it increased from 0.7749 to 0.8701 between 2005 and 2010) indicates that the network is not segmented between sectors but is in fact a basis for a broad European business community across sectors.

With these basic statistics we confirm and further corroborate that the network of interlocking directorates in Europe is indeed increasing, and that this increase is not confined to national network but includes an increasing set of edges between firms with different domiciles: genuine European board interlocks.

### Assortativity and mixing patterns

**Figure 3:**
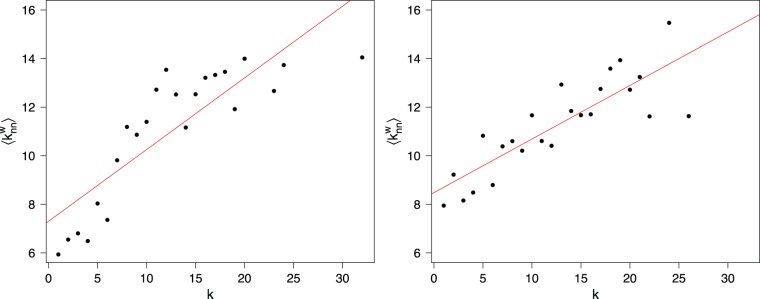
Assortative mixing in the boards projection in 2005 (Left) and 2010 (Right). The plots show the behavior of the weighted average degree of a node's nearest neighbors as a function of the node degree. Thin red lines display linear regressions: slope coefficients are 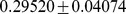 and 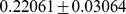 for 2005 and 2010, respectively, whereas the regression 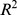 is 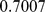 and 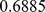.

In complex networks, properties of connected nodes can be positively correlated, negatively correlated, or uncorrelated. For example, in most technological networks, the degree of neighboring nodes is negatively correlated (disassortive mixing), but in most social networks degree is positively correlated (assortative mixing) [36], [37]. The European network of interlocking directorates is indeed degree assortative both in 2005 and in 2010, i.e. a firm with a high number of connections tends to be connected to others highly connected firms, and conversely (Fig. 3). Newman's 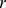 coefficients are given in Table 1.

The interaction with similarly connected corporations reinforces the dividing lines and contributes to a hierarchy in the network. Well-connected firms flock together in dense parts of the network while less connected firms predominantly have ties with other less connected firms as well. Interestingly, there is a significant decrease in 2010 from 2005 in Newmans 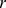. Firms are significantly less prone to connect to others with the same connectivity. This suggests that the European network is developing towards a less hierarchical, more inclusive social structure. In Figures S2 and S3 in File S1 we provide a 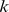-core visualization of the network [38], which confirms that the 2010 network is less hierarchically structured than the 2005 one.

A useful manner in which we can further investigate the transnational properties of the network is to consider how inclined firms are to mix with respect to their country of origin as far as their boards are concerned. We call this geographic assortativity and it represents another kind of correlation that can be investigated by means of the corresponding assortativity coefficient. From this point of view, as we can expect, firms are still prone to relate to firms within their own country of residence. However, this assortativity score decreases from 0.5872 in 2005 to 0.5303 in 2010. At the same time, the inclination of firms to maintain interlocks with firms in the same sector increased from 0.000647 in 2005 to 0.02660 in 2010. The effect remains limited though, and the geographical pattern still is more important than the economical one, i.e. firms tend to share more directors with other firms from the same country than with firms of the same sector.

### Central actors analysis

A key characteristic of the network topology are its organizing pillars or most central nodes. We consider the largest connected component of the weighted board projections for 2005 and 2010, and perform a key-actor analysis. Network centrality is often measured by counting the degree: the number of edges per node. This only takes into account the direct neighborhood (ego network) of a node and not its position in the wider network structure. Therefore it does not help us to distinguish between a node that is highly central within one dense pocket of the network and one that brokers different parts of the network. In order to overcome this problem we combine two well-established centrality measures that take into account the wider network structure (see Methods section). First, eigenvector centrality tries to capture the idea that the more central the neighbors of a vertex are, the more central that vertex is. This measure is typically expressed in terms of the eigenvectors of the network adjacency matrix, whose eigenvalues can be interpreted as indices of nodes accessibility [39]. Since the network is degree assortative, there will be subgraphs where firms with large degree centrality are mutually connected. Membership of these “thick pockets” in the network is indicated by eigenvector centrality. Through Bonacich's eigenvector centrality we gauge the status, or “rank” of a given firm in the network.

**Figure 4:**
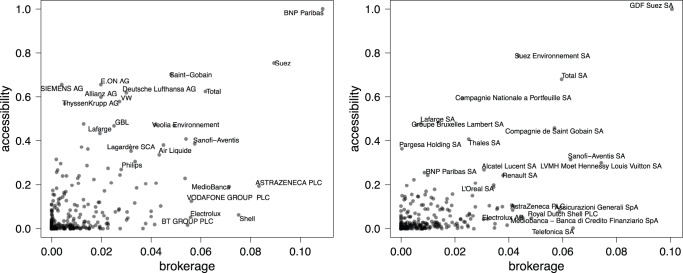
Centrality measures on the boards projection in 2005 (Left) and 2010 (Right). Each point in the plane corresponds to a company; 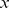 and 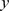 coordinates define its betweenness (here renamed brokerage), and eigenvector centrality (here called accessibility) scores, respectively. Due to readability reasons, only those who rank higher are labeled (refer to Tables 2 and 3).

Betweenness centrality on the other hand measures the extent to which a firm brokers distant parts of the network. High brokerage may suggest a central position in building the European network by connecting different communities. Tables 2 and 3 rank the more central firms according to each metric for both years, Fig. 4 displays the firms on both eigenvector and betweenness centrality measures. We can distinguish three main regions in the plots. The upper right area towards the corner features firms that are connected to well-connected others and have a high betweenness at the same time; they can be called “hubs”.

**Table 2:** Top 10 ranked firms with respect to three different measures of centrality – Year 2005.

Rank	Degree	Eigenvector	Betweenness
1	BNP Paribas	BNP Paribas	BNP Paribas
2	Suez	Suez	Suez
3	Allianz AG	Saint-Gobain	Astrazeneca PLC
4	Total	Siemens AG	Shell
5	Veolia Environnement	E.ON AG	MedioBanca
6	Deutsche Lufthansa AG	Total	Total
7	Saint-Gobain	Deutsche Lufthansa AG	Sanofi-Aventis
8	Lagardère SCA	Allianz AG	Vodafone Group PLC
9	Sanofi-Aventis	VW	Electrolux
10	Astrazeneca PLC	ThyssenKrupp AG	BT Group PLC

**Table 3:** Top 10 ranked firms with respect to three different measures of centrality – Year 2010.

Rank	Degree	Eigenvector	Betweenness
1	GDF Suez SA	GDF Suez SA	GDF Suez SA
2	Total SA	Suez Environnement SA	Moet Hennessy Vuitton SA
3	Sanofi-Aventis SA	Total SA	Telefonica SA
4	Comp. de Saint Gobain SA	Comp. Nat. a Portfeuille SA	Sanofi-Aventis SA
5	BNP Paribas SA	Lafarge SA	Total SA
6	Moet Hennessy Vuitton SA	Groupe Bruxelles Lambert SA	Assicurazioni Generali SpA
7	Thales SA	Comp. de Saint Gobain SA	Comp. de Saint Gobain SA
8	AstraZeneca PLC	Thales SA	Royal Dutch Shell PLC
9	Comp. Nat. a Portfeuille SA	Pargesa Holding SA	Electrolux AB
10	Renault SA	Sanofi-Aventis SA	Mediobanca

The upper left side of the figures groups firms that are directly connected to central actors but are not themselves on the main shortest paths. Firms lying in the lower right region of the figures are the “brokers”, i.e. the firms that stand on the shortest paths and bridge different parts of the network but not connected to well-connected others. French firms stand out both in terms of eigenvector as well as betweenness centrality. In 2005 there is still a batch of German firms in the top left quadrant of Fig. 4 (left), but by 2010 the top half of the figure is occupied by French firms only (Fig. 4 right). The decrease in hierarchy as already noted in the previous paragraphs is also illustrated by the more concentrated and overall lower scores on eigenvector centrality in 2010 as compared to five years before. Finally, there is the group of firms with low eigenvector scores (below .25) but high betweenness (above .25). These firms are perhaps the more transnational oriented ones, partly also because their home countries have smaller business communities and hence less opportunities to interlock. Tables 2 and 3 further reveal that the core of the more central firms is not entirely stable between 2005 and 2010: firms move in and out the most central areas.

**Figure 5:**
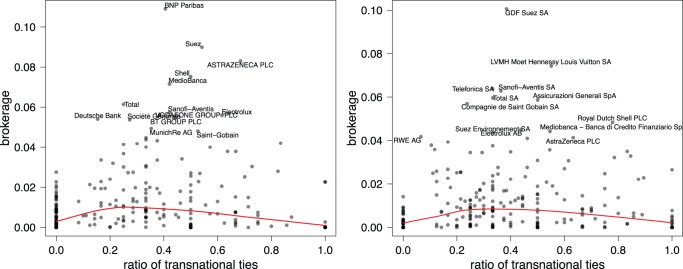
Brokerage against transnational character in 2005 (Left) and 2010 (Right). Each point in the plane corresponds to a company; 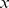 and 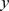 coordinates define its normalized node betweenness (brokerage) and its ratio of transnational edges, respectively. For readability reasons, only company names in the upper 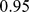-quantile of the betweenness distribution are shown.

All in all, the core of the network is not dominated by one country but transnationally composed. This brings us to the issue how transnationally oriented the central firms themselves are. For this, we computed the ratio of transnational edges per firm as an indicator of the transnational character. Figure 5 plots this ratio against the betweenness centrality. It shows that those firms that broker distant parts of the network are indeed part of the transnational network. However, the relation has an inverted u-shape where the firms which are most transnational have relatively low levels of brokerage. The top brokers are not the most transnational firms. Rather, the top brokers combine a strong national position with a strong European position; they are linchpins between the national and the European. In that sense, organizing pillars of the European network still build on national business communities. At the same time there is a set of firms that are highly transnational, but do not occupy key broker positions in the network. Again, this is probably due to smaller board size, which limits brokerage opportunities and increases the probability for high ratios.

### Community structure

**Figure 6:**
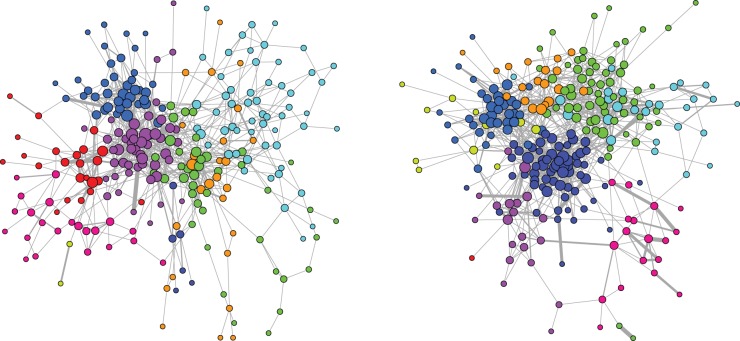
Communities in the largest connected components of firms projection in 2005 (Left) and 2010 (Right). The size of a node is proportional to the square root of its number of connections, the thickness of an edge is proportional to the number of shared directors between the connecting boards. Colors refer to the best-found community partition, whose weighted modularity score is 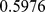 for 2005 and 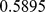 for 2010.

Finally, we probe the underlying community structure of the emerging European network of interlocking directorates. Community detection finds a subdivision into 10 subgroups for both networks (Fig. 6). Modularity scores for those clusterings in 0.5976 for 2005 and 0.5895 in 2010, and provide evidence of a significant network partitioning. Indeed, performing the statistical tests described in Sect. Methods, shows that both observed values are more than 35 st. dev. away from the expected modularity score of the null model, supporting the discovery of a meaningful community structure (see Figure S4 in File S1). On the other hand, although in all tests the network of 2010 appears to be slightly less modular then the one of 2005, we don't have enough support to claim that this difference is statistically significant. Tables 4 and 5 report the distribution of countries within those communities. A closer look at the table reveals that the ten communities can be easily related to sets of firms with similar domicile that are dominant. We labeled the communities according to the most dominant nationality present. The community detection approach thus suggests that still in 2010, the European network of interlocking directorates rests firmly on its national bases. Once again, the geographical factor still plays a dominant role. Indeed, when considering the natural clustering given by the country of origin, we obtain a weighted modularity score of 0.5159 in 2005 and of 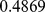 in 2010. Albeit lower, it shows that geography still plays an important role in building the communities. In line with the observations above, a similar grouping taking sectors into account, gives no indications that board interlocks cluster within sectors. In fact, the scores given by the division into sectors are 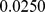 and 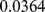 for 2005 and 2010, respectively, denoting a very low modularity of such a partitioning (see Tables S1 and S2 in File S1 for the details about the distribution of sectors across communities). These results are coherent with the analysis of mixing patterns.

**Table 4:** Country distribution of the best-found subdivision into communities – Year 2005.

Community	1	2	3	4	5	6	7	8	9	10	
	Italy	Sweden	Italy2	UK- Bel.	Netherlands	UK	Germany	Switzerland	France	Spain	# communities
Country		.									
Austria	2	.	.	.	.	.	1	.	.	.	2
Belgium	.	1	.	6	.	.	.	.	5	.	3
Denmark	.	3	.	.	.	.	.	.	.	.	1
Finland	.	5	.	.	.	.	.	.	.	.	1
France	4	.	.	.	.	.	1	1	37	.	4
Germany	.	.	.	.	.	.	34	.	.	.	1
Greece	.	.	.	.	.	.	.	.	1	.	1
Ireland	.	.	.	.	.	1	.	.	1	2	3
Italy	14	.	2	.	.		.	.	.	7	3
Netherlands	.	.	.	.	17	1	1	.	.	.	3
Norway	.	2	.	.	.		.	.	.	.	1
Portugal	.	.	.	.	.		.	.	.	1	1
Spain	3	.	.	.	.		.	.	.	11	2
Sweden	.	11	.	.	.		.	.	1	.	2
Switzerland	.	1	.	.	1		3	5	3	1	6
UK	.	3	.	7	10	54	.	.	1	.	2
# of firms	23	26	2	13	28	56	40	6	49	22	265
# of countries	4	7	..	2	3	3	5	2	7	5	3.9
# firms main country	14	11	..	7	17	54	34	5	37	11	21.11
# firms others	9	15	..	6	11	2	6	1	12	11	8.11
% firms others	39.13	57.69	..	46.15	39.29	3.57	15.00	16.67	24.49	50.00	32.44

**Table 5:** Country distribution of the best-found subdivision into communities – Year 2010.

Community	1	2	3	4	5	6	7	8	9	10	
	Italy2	Netherlands	Switzerland	UK	Iberian	Sweden	Germany	France	Italy	Spain	# communities
Country	.										
Belgium	.	.	.	4	.	.	.	5	.	.	2
Denmark	.	.	.	1	.	4	.	.	.	.	2
Finland	.	5	.	.	.	.	.	.	.	.	1
France	.	.	1	1	.	.	.	46	2	1	5
Germany	.	.	.	.	.	1	29	.	1	.	2
Ireland	.	1	.	1	.	.	.	1	.	.	4
Italy	1	.	.	.	.	.	1	2	12	3	5
Luxembourg	.	.	.	.	.	.	.	3	.	.	1
Netherlands	.	11	.	3	.	.	1	1	.	.	4
Norway	.	.	.	.	.	4	.	.	.	.	1
Portugal	.	.	.	.	1	.	.	.	2	2	3
Spain	.	.	.	.	1	.	1	.	1	12	4
Sweden	.	.	.	.	.	17	.	.	.	.	1
Switzerland	.	.	10	2	.	1	4	2	2	.	6
UK	.	2	.	49	.		1	3	.	.	4
# of firms	1	19	11	61	2	27	37	63	20	18	259
# of countries	.	4	2	7	.	5	6	8	6	4	4.5
# firms main country	.	11	10	49	.	17	29	46	12	12	23.25
# firms others	.	8	1	12	.	10	8	17	8	6	8.75
% firms others	.	42.11	9.09	19.67	.	37.04	21.62	26.98	40.00	33.33	2.73

Based on the number of countries per community, we can distill a number of useful statistics such as transnational orientation of the communities and the countries (Tables 4 and 5). Thus, in 2005 we find that strongly transnational oriented communities are the Swedish (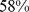 of non-Swedish firms in the community) and the Spanish (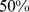 non-Spanish firms in the community). The Swedish community combines this with a wide reach: it connects to seven different countries compared to five for the Spanish community. The French and German communities also have a wide reach, which is not surprising given their large size. Switzerland shows as the country with the most connections to the ten communities, followed by the markedly larger UK. One cluster is bi-national and connects Belgian with British firms. (The Italy2 community contains two Italian firms that are strongly connected to each other but have only one link to the rest of the network; see Fig. 6 where these two cases are highlighted).

On average countries belong to 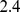 communities in 2005 but increase this to 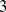 by 2010. Although there are still clearly national oriented communities, the average number of countries per community increases from 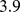 to 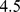. The findings reveal a development where the communities are more comparable in terms of international orientation. The high scores of the Spanish and Swedish communities drop to 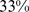 and 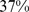 while low scoring communities in 2005 such as the UK and Germany increase the international reach of their community by 2010. Switzerland remains the country that partakes in the most communities (six), while the Dutch community has the largest share of foreign members. These two small internationalized countries are becoming hubs in the network. At the same time the dominant economic powers of France, Germany, and the UK are more transnationally oriented in their business community.

**Figure 7:**
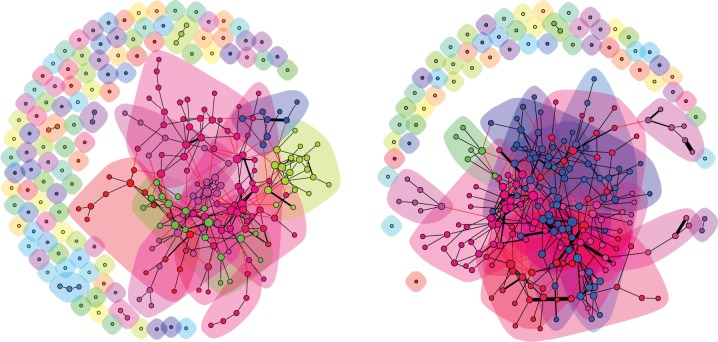
Communities in the subgraph of firms projection in 2005 (Left) and 2010 (Right) considering transnational links only. The size of a node is proportional to the square root of its number of connections, the thickness of an edge is proportional to the number of shared directors between the connecting boards. Colors refer to the best-found community partition, whose weighted modularity score is 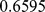 for 2005 and 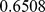 for 2010. Note that the largest connected component covers 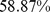 of the transnational subgraph in 2005, and 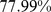 in 2010.

It is of interest to try to understand what is the extent of the community structure that is attributable to transnational ties only. To do that, we remove from the graphs the links between companies of the same country. The induced subgraphs thus obtained are both clustered into separate components. Notably, though, the relative size of the largest one, the giant component, is much higher in 2010 than in 2005 (see Fig 7). This adds further evidence of the increased transnational character in the boards interlocks. On the other hand, although the communities in these new graphs are rather well defined, we found no evidence of clear relationships with respect to sector or country of origin.

## Discussion

At the peak level of governance, firms are part of social elite networks that tie together the key corporate decision-making bodies: the boards of directors. In this paper we show that the remarkable absence of a growing transnational network of interlocking directorates following the years of economic and financial globalization in the late 20th century may now have come to an end. Within Europe, the network interlocking directorates is steadily growing. This is even more remarkable given the steady decrease in national networks of board interlocks across the globe over the past decades [13], [35].

The key issue we addressed is the extent to which this network can be said to be transnational, as opposed to a superstructure that rests on national bases. The reliable method of community detection that we applied has been a helpful tool in addressing this issue. Community detection rendered a number of robust subsets of the network. A membership analysis of the communities revealed that all had a distinct national character. The European network of interlocking directorates still stands on the shoulders of national business communities. At the same time, the communities are transnational in that they include firms from other countries as well. And from 2005 to 2010 the communities on average become more transnationally composed. Community detection is therefore a useful and also promising tool for research into interlocking directorates in particular and social networks in general. The results of the community detection, key actor and assortativity analyses are mutually enforcing. They show that at the level of corporate governance, Europe is getting closer. A European corporate elite may well be in the making. A key observation is that over time, the network becomes less hierarchic and more equally distributed. This takes place both if we consider network metrics such as the distribution of eigenvector centrality, but also if we look at the transnational composition of the communities. In general we see that the findings and metrics in 2010 are less pronounced and more equal than five years before. As it develops, the European network of interlocking directorates depends less and less on particular firms, persons or communities as its underpinning. If this continues, we may well see a genuine European network emerging soon, where well-connected European firms do away with their former strong ties in national business communities.

At the same time, we showed that geography plays an important role in wiring the social network of corporate boards. Distance is cumbersome for attending board meetings but reflects cultural differences as well. In this the board network is different from European ownership network where the structure is only partially explained by geography [40]. While geography does play a role, we find no evidence that the network clusters around particular economic sectors. The effect of geography was larger in 2005 than five years later, which further illustrates the move towards transnational interlocks.

Our results also provide an exceptional peek into the European orientation of big business. The process of European Unification has always been strongly supported by large industrial conglomerates and business interests. The common market and later the monetary union were seen as in the interest of European business in general. Over the past decade however the project of European Unification has received more and more critique. In the light of the political turmoil about the Unions future, it is telling that European corporations increasingly link with each other across European borders. France and also Germany play a key role in this network, reflecting both their political position as drivers of European unification as their economic and industrial position within Europe. But interestingly so, a number of small countries emerge as brokers within the European network. Countries as Switzerland and the Netherlands have always been friendly environments for multinational corporations and internationally oriented in general. Indeed, within the political playing field within Europe it is not uncommon for small countries to receive key political positions (the EU presidency for Belgium, Portugal with the chair of the European Commission). Our analysis shows that this is equally true within the network of corporate interlocking directorates.

Whether the European network will continue to strengthen after 2010 is an empirical question. The effects of the financial and economic crisis from 2008 onward are only partly reflected in our findings on 2010. We should continue to observe the developments of the European network. For now it seems the only market where interlocking directorates are increasing. Future steps that seem apt are to compare the European network to other regional networks such as in the USA, and further compare the properties of the European network to that of several national business communities. The community detection algorithm we applied here will serve as a helpful tool. The growing availability of data makes it possible to investigate transnational elite networks of corporate board members on a global scale. Community detection is especially promising here because it allows us to identify in a simple and elegant manner where the genuine transnational regions in corporate networks are located.

## Supporting Information

File S1 **Figure S1, Degree (Left) and strength (Right) distributions.** The empirical distributions are of the cumulative complementary type. The scale is logarithmic on both axis; the insets show the same plot on a semi-logarithmic scale. Round black points refer to year 2005, triangular red points to year 2010, as in the legend. **Figure** **S2, k-core decomposition of the boards projection in 2005.** Node size scales with degree, color and spatial arrangement depend on the shell index (see legends on both sides). **Figure** **S3., k-core decomposition of on the boards projection in 2010.** Node size scales with degree, color and spatial arrangement depend on the shell index (see legends on both sides). **Figure** **S4, Q-Q plot of modularity on the randomized networks of 2005 (Left) and 2010 (Right).** Both plots show no significant deviation from the theoretical quantiles of a normal distribution. Picture insets report the density plots of the modularity data on those randomized networks. The mean and standard deviation are, respectively, 0.4201 and 0.0048 for 2005, and 0.4134 and 0.0046 for 2010. In both cases, the modularity score obtained from the real networks is far away from the expected value of the respective null-model and can be considered statistically significant. **Table S1, Sector distribution of the best-found subdivision into communities – Year 2005. Table** **S2,** **Sector distribution of the best-found subdivision into communities – Year 2010.**
(PDF)
