# Supplementary material for: The Community Structure of the European Network of Interlocking Directorates 2005–2010
Source: PLoS One. 2013 Jul 19;8(7):e68581. doi: 10.1371/journal.pone.0068581 (PMC3716815; doi:10.1371/journal.pone.0068581)
Supplement: File S1 — Figure S1, Degree (Left) and strength (Right) distributions. The empirical distributions are of the cumulative complementary type. The scale is logarithmic on both axis; the insets show the same plot on a semi-logarithmic scale. Round black points refer to year 2005, triangular red points to year 2010, as in the legend. Figure S2, k-core decomposition of the boards projection in 2005. Node size scales with degree, color and spatial arrangement depend on the shell index (see legends on both sides). Figure S3., k-core decomposition of on the boards projection in 2010. Node size scales with degree, color and spatial arrangement depend on the shell index (see legends on both sides). Figure S4, Q-Q plot of modularity on the randomized networks of 2005 (Left) and 2010 (Right). Both plots show no significant deviation from the theoretical quantiles of a normal distribution. Picture insets report the density plots of the modularity data on those randomized networks. The mean and standard deviation are, respectively, 0.4201 and 0.0048 for 2005, and 0.4134 and 0.0046 for 2010. In both cases, the modularity score obtained from the real networks is far away from the expected value of the respective null-model and can be considered statistically significant. Table S1, Sector distribution of the best-found subdivision into communities – Year 2005. Table S2, Sector distribution of the best-found subdivision into communities – Year 2010. (PDF) [file pone.0068581.s001.pdf]

The Community Structure of the European Network of Interlocking Directorates 2005-2010. Supporting Information

Eelke M. Heemskerk[1], Fabio Daolio[2], Marco Tomassini[3,*]

1 Eelke M. Heemskerk Amsterdam Institute for Social Science Research, University of Amsterdam, The Netherlands

2 Fabio Daolio Department of Information Systems, University of Lausanne, Switzerland

3 Marco Tomassini Department of Information Systems, University of Lausanne, Switzerland

∗ E-mail: fabio.daolio@unil.ch

Supporting Figures

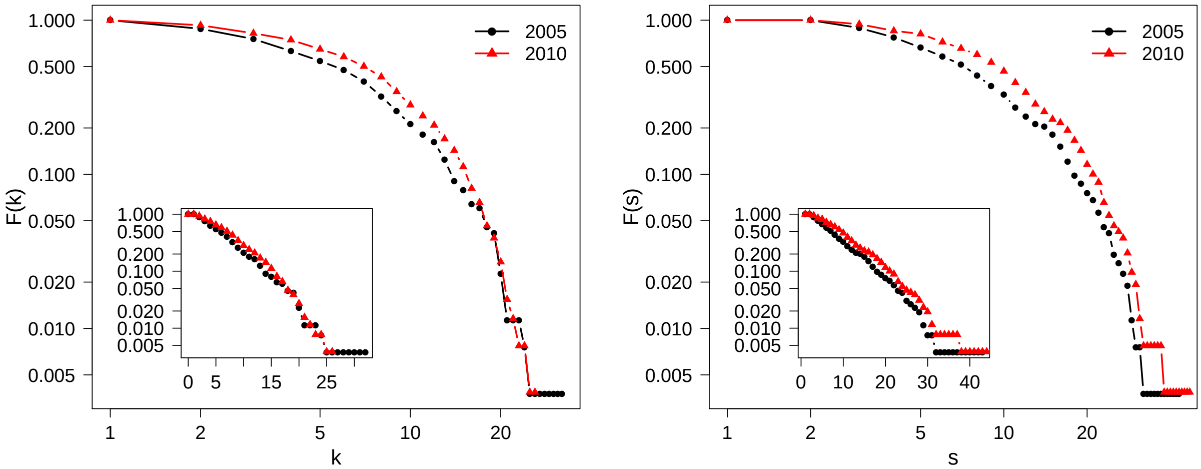

Figure S1. Degree (Left) and strength (Right) distributions. The empirical distributions are of the cumulative complementary type. The scale is logarithmic on both axis; the insets show the same plot on a semi-logarithmic scale. Round black points refer to year 2005, triangular red points to year 2010, as in the legend.

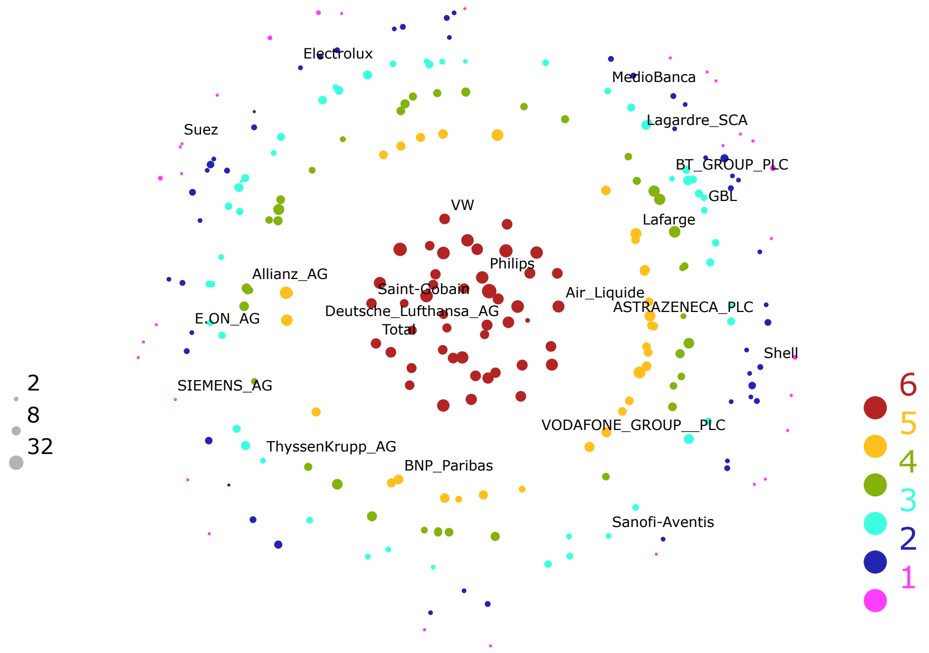

Figure S2. k-core decomposition of the boards projection in 2005. Node size scales with degree, color and spatial arrangement depend on the shell index (see legends on both sides).

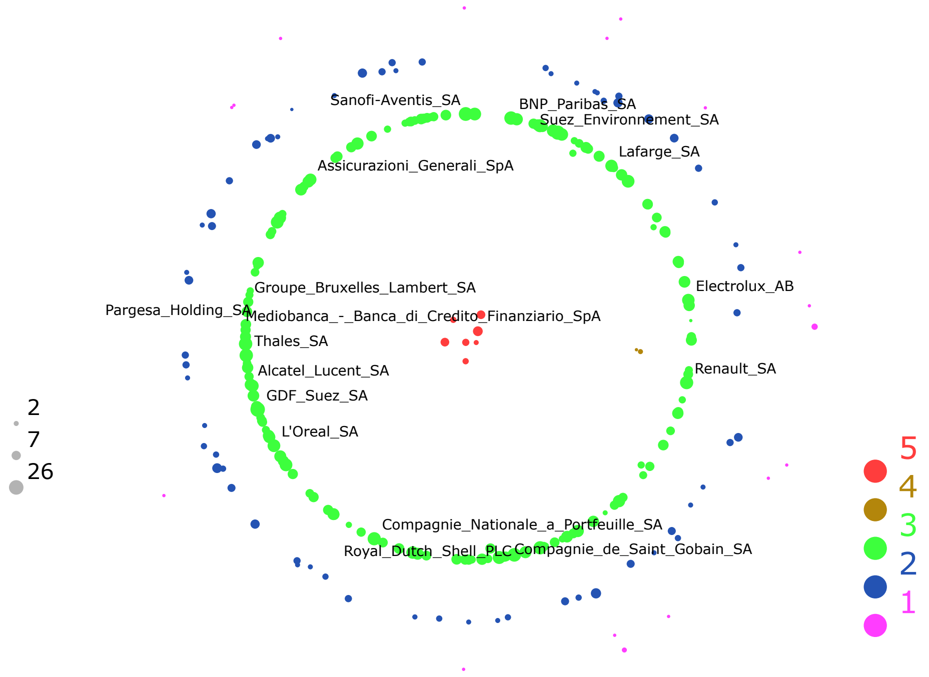

Figure S3. k-core decomposition of on the boards projection in 2010. Node size scales with degree, color and spatial arrangement depend on the shell index (see legends on both sides).

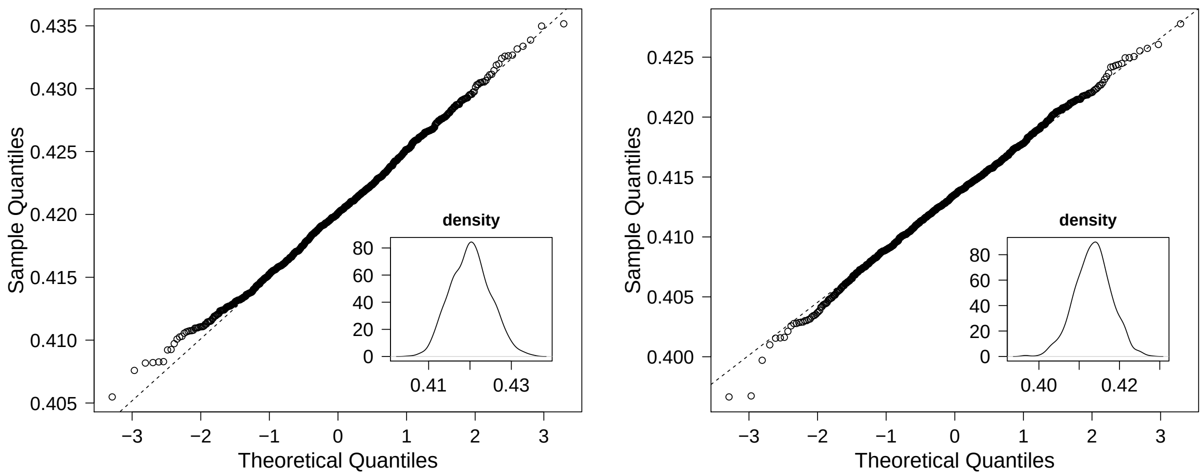

Figure S4. Q-Q plot of modularity on the randomized networks of 2005 (Left) and 2010 (Right). Both plots show no significant deviation from the theoretical quantiles of a normal distribution. Picture insets report the density plots of the modularity data on those randomized networks. The mean and standard deviation are, respectively, 0.4201 and 0.0048 for 2005, and 0.4134 and 0.0046 for 2010. In both cases, the modularity score obtained from the real networks is far away from the expected value of the respective null-model and can be considered statistically significant.

Supporting Tables

Table S1. Sector distribution of the best-found subdivision into communities - Year 2005

Community	1	2	3	4	5	6	7	8	9	10
Sector										
Aerospace & Defense	1	·	·	·	1	1	·	·	2	·
Automotive	·	·	·	·	·	·	5	·	3	1
Banks	11	5	·	1	3	9	6	1	5	3
Chemicals	·	·	·	1	2	1	4	·	1	·
Communications	·	·	·	·	·	1	·	·	3	·
Construction	·	·	·	1	·	·	·	·	4	3
Consumer Goods & Retail	2	·	·	5	2	10	3	1	5	2
Electronics	·	·	·	·	1	·	·	·	1	·
Energy	3	1	·	·	3	3	·	·	3	4
Financials	3	3	1	·	3	4	4	1	7	·
Food	·	·	·	1	3	3	1	1	1	·
Healthcare	·	·	·	·	1	·	1	1	3	1
Industry & Services	·	8	·	·	1	1	5	·	2	2
IT	·	·	·	·	·	·	2	1	·	·
Media and Publishing	·	1	1	1	3	4	·	·	5	·
Mining	·	1	·	·	·	4	1	·	·	·
Pharmaceuticals	·	2	·	1	·	2	3	·	·	·
Real Estate	1	·	·	·	1	2	·	·	·	·
Services	·	·	·	1	1	4	·	·	1	·
Telecom	1	4	·	1	1	3	1	·	·	4
Transport and Logistics	·	·	·	·	1	2	2	·	2	1
Utilities	1	1	·	·	1	2	2	·	1	1

Table S2. Sector distribution of the best-found subdivision into communities - Year 2010

Community	1	2	3	4	5	6	7	8	9	10
Sector										
Aerospace & Defense	·	·	·	2	·	·	1	2	·	1
Automotive	·	·	·	·	·	1	3	3	1	·
Banks	1	1	1	4	·	4	5	6	5	1
Chemicals	·	2	2	·	·	1	2	2	·	·
Communications	·	·	·	·	·	1	·	4	·	1
Construction	·	·	1	1	·	2	·	5	·	3
Consumer Goods & Retail	·	2	1	10	·	2	3	5	3	·
Electronics	·	1	·	·	1	·	·	4	·	2
Energy	·	1	·	3	·	2	·	4	4	4
Financials	·	3	·	5	·	3	6	6	2	1
Food	·	·	1	5	·	·	·	3	·	·
Healthcare	·	1	3	1	·	1	1	3	·	·
Industry & Services	·	3	1	2	·	5	5	1	2	·
IT	·	·	·	·	·	·	2	2	·	·
Media and Publishing	·	·	·	5	·	·	·	2	1	·
Mining	·	·	·	5	·	·	2	2	·	·
Pharmaceuticals	·	·	·	4	·	1	1	·	·	·
Real Estate	·	·	·	3	·	·	·	1	·	·
Services	·	·	1	5	·	·	·	2	·	·
Telecom	·	3	·	3	·	3	1	·	1	2
Transport and Logistics	·	1	·	·	·	1	2	3	1	·
Utilities	·	1	·	3	1	·	3	3	·	3
